# Mortality and causes of death among violent offenders and victims-a Swedish population based longitudinal study

**DOI:** 10.1186/1471-2458-12-38

**Published:** 2012-01-17

**Authors:** Marlene Stenbacka, Tomas Moberg, Anders Romelsjö, Jussi Jokinen

**Affiliations:** 1 Addiction Center Stockholm, Karolinska University Hospital Solna, Building Z8, 171 76 Stockholm, Sweden; 2 Department of Public Health Sciences, Division of Social Medicine, Karolinska Institute, Norrbacka Building, 171 76 Stockholm, Sweden; 3 Department of Clinical Neuroscience, Karolinska Institute, Karolinska University Hospital Solna, 171 76 Stockholm, Sweden

## Abstract

**Background:**

Most previous studies on mortality in violent offenders or victims are based on prison or hospital samples, while this study analyzed overall and cause specific mortality among violent offenders, victims, and individuals who were both offenders and victims in a general sample of 48,834 18-20 year-old men conscripted for military service in 1969/70 in Sweden.

**Methods:**

Each person completed two non-anonymous questionnaires concerning family, psychological, and behavioral factors. The cohort was followed for 35 years through official registers regarding violent offenses, victimization, and mortality. The impact of violence, victimization, early risk factors and hospitalization for psychiatric diagnosis or alcohol and drug misuse during follow up on mortality was investigated using Cox proportional hazard regression analyses.

**Results:**

Repeat violent offenses were associated with an eleven fold higher hazard of dying from a substance-related cause and nearly fourfold higher hazard of dying from suicide. These figures remained significantly elevated also in multivariate analyses, with a 3.03 and 2.39 hazard ratio (HR), respectively. Participants with experience of violence and inpatient care for substance abuse or psychiatric disorder had about a two to threefold higher risk of dying compared to participants with no substance use or psychiatric disorder.

**Conclusions:**

Violent offending and being victimized are associated with excess mortality and a risk of dying from an alcohol or drug-related cause or suicide. Consequently, prevention of violent behavior might have an effect on overall mortality and suicide rates. Prevention of alcohol and drug use is also warranted.

## Background

Violence is a serious public health concern, especially among young people. Previous studies have shown that violent and criminal offenders have increased mortality [1-4]. Tikkanen et al. found four times higher mortality among non-psychotic violent male offenders with alcohol problems than among controls from the general population [4]. Alcohol-related diseases, poisonings, and injuries caused by violence accounted for 49% of deaths in the violent offender group and 21% in the control group. Other longitudinal studies have presented similar results [1,5,6]. It has been discussed whether alcohol misuse is a cause of violence or a consequence of a criminal lifestyle, or whether these behaviors are mainly related to common background factors, and the results vary between studies [7-10]. People with psychiatric disorders evince an excess mortality of both natural and unnatural causes [11].

Understanding the link between psychiatric disorders and violent offending requires consideration of its association with history of violence, substance abuse and stressful life events [12]. Persons with mental disorders have an elevated risk for violent victimization [13,14]. A random sample of 936 psychiatric outpatients in Chicago was 11 times more likely to be victimized by violence than the comparison group of 32,000 participants in the National Crime Victimization Survey [15]. Results from another study showed that individuals with mental disorders, alcohol and marijuana dependence experienced more often physical assault [14]. In a study of Silver et al., individuals with mental disorders were more likely to experience both victimization and convictions due to violence than individuals with no mental illness [14,16,17].

There is evidence about overlapping between offending and victimization due to violence concerning early social characteristic and behaviors [18]. It has been reported that being victimized in itself is a risk factor for repeated victimization in the future [18,19]. Likewise, a two year follow-up study by Shaffer and Ruback showed that earlier violent victimization, being male, substance abuse and violent offending were predictors for violent revictimization at follow-up [20]. Early emotional and behavioral problems seem to be common risk factors for both bullying and victimization among children and adolescents [21]. In existing studies, the samples are usually drawn from the criminal justice system or hospitals, which may not be representative of people in general [11,22].

Few studies have examined the violent offending-violent victimization overlap and mortality pattern in a large population based cohort. There is a paucity concerning the relationship between violent offence and victimization in relation to mortality and causes of death in general longitudinal population-based studies [16].

In this representative, nationwide, large general population study we followed 48,834 Swedish conscripts from the age of 18 up to the age of about 53, to investigate whether violent offending or being a victim of violent offending increases mortality, and if this risk is higher among those with repeated violent offending. We also investigated whether family, social and psychological factors during late adolescence modified the relationship between violent offending and death.

The specific research questions in this study were:

i) Do violent offenders, victims of violence, and those who are both offenders and victims, with or without substance misuse or psychiatric disorder, have increased mortality compared to other individuals?

ii) Do mortality and causes of death differ between these three categories of individuals after controlling for early risk factors?

iii) Do violent recidivists differ from single offenders regarding mortality and causes of death?

## Methods

### Participants

From 1 July 1969 to 30 June 1970, 50465 men 18-20 years were conscripted for military service in Sweden. Only a small part (2-3%) was exempted from the military service mainly because of a congenital psychiatric or physical disorder or severe handicap. Most of the conscripts were born in 1949 (6%), 1950 (18%) and 1951 (75%) and in order to get as homogenous group as possible according to age, we included 48834 conscripts born 1949-51 and followed them until 2004. Their mean age at the end of the follow-up was about 53 years of age.

### Measures

Each participant completed two non-anonymous questionnaires. The first included questions about family and social background, behavior, and health and the second included questions about cigarette smoking, and alcohol and drug use. The questionnaires have been found to have sufficient validity for epidemiological studies [23-26]. Each participant was seen by a trained military psychologist for a structured interview and assessed on a series of variables based on questionnaire and interview data. The measures were assessed on a 1-5-point Likert scale (5 being the highest), yielding a distribution corresponding to 1 = 7, 2 = 24, 3 = 38, 4 = 25 and 5 = 7%. The ratings of the psychologists were regularly checked for inter-rater reliability in order to check good quality [26]. The conscript was referred to a psychiatrist if any psychiatric disorder was suspected, or reported by the conscript. An eventual diagnosis was coded according to ICD-8 [25,26].

Based on items from the questionnaires and earlier studies of risk factors for violence and mortality, we included 19 variables associated with both lifetime violent offending and mortality. After bivariate Cox proportional regression analysis, we selected ten significant variables as confounders for multivariate analyses. Variables included were: parents' divorce (yes vs. no), any previous contact with the police or juvenile authorities (yes vs. no), conduct problems at school (yes vs. no), ever run away from home (yes vs. no) and smoking (≥ 10 cigarettes/day vs. < 10 cigarettes/day or none). Also included were problem drinking (yes vs. no, with 'yes' defined as consumption of ≥ 210 g pure alcohol per week, being intoxicated often, and/or having been apprehended for public drunkenness on at least one occasion) and drug misuse, defined as having used illicit drugs 10 times or more or having used drugs intravenously one or more times vs. less than ten times or no use.

Included psychological variables were emotional control (a summary of assessed mental stability, emotional capacity, and tolerance to stress and frustration) and social maturity (feeling responsibility for activities by other persons, and having a sense of independence, and a degree of social extroversion). The two variables were assessed on the five point scale, where 1 = very bad; 2 = bad; 3 = moderate; 4 = good; 5 = very good, with the categorizations emotional control 1-2 vs. 3-5, and social maturity 1-2 vs. 3-5. Psychiatric diagnosis at conscription was defined as: yes (one or more diagnoses) vs. no.

### Follow up data

The cohort was followed through official registers and was linked at Statistics Sweden via the unique personal number for each individual in the cohort, which then was replaced with an individual serial number making the data anonymous to the research group, after approval of Karolinska Institute Research Ethics Committee (Dnr 2007/174-31, Dnr 2008/1086-31/5).

### Hospital data

Data from the National Hospital Register were used to identify inpatient care with violence, alcohol and drug use according to ICD-8 and ICD-9 from 1987 and ICD-10 from 1997 onwards.

The National Hospital Register includes details of inpatient care, and has covered all public hospitals in Stockholm and Uppsala County since 1972, 85% of all Swedish public inpatient care stays since 1983, and about 98-99% of such care stays since 1987. The ICD classifications for hospitalization and mortality were: Violence - ICD-8 and ICD-9: E960-E969; ICD-10: X85-X99, Y00-Y09. Drug misuse - ICD-8: 304 and 965.0; ICD-9: 304, 965A, 968 F, 969 G and 969H; and ICD-10: F11-12, F14, F15, F16, F18, F19, O35.5, P04.4, T40.0-T40.3, T40.5-T40.9, T43.6, Z71.5, and X42. Alcohol misuse - ICD-8: 291, 303, 571.00, 571.01 and 980; ICD-9: 291, 303, 305A, 357 F, 425 F, 535D, 571A-571D and 980; and ICD-10: E24.4, F10, G31.2, G62.1, G72.1, I42.6, K29.2, K70, K86.0, O35.4, P04.3, Q86.0, T51, X45, Y91, Z50.2 and Z71.4. Psychiatric disorders (except alcohol and drug diagnoses): ICD8, and ICD9 290-319 and ICD10: F00-F69.

### Mortality data

Mortality data came from the Cause of Death Register, which covers more than 99% of all deaths occurring in Sweden and is based on information from death certificates. Underlying causes of death are classified according to ICD-8, ICD-9 and ICD-10 as for the hospital register. One underlying cause of death is given on each death certificate, although contributing causes can be added.

### Criminal records

Data from the national Crime Register were used to identify date, type, and number of criminal offense. The Crime Register contains information on all convictions in Sweden from 1966 onwards. Serious violence was defined as: homicide, manslaughter and assault. Violent offense were categorized as follows: conviction for violence (at least one vs. none) and violent recidivism (two or more convictions vs. none).

### Statistical methods

Cox proportional bivariate and multivariate regression analyses were used to calculate the hazard ratios (HRs) in the three groups of violence and all other conscripts for mortality and causes of death from 1 January 1970 up to the time of death or to the end of the observation period (December 31, 2004). The person times were calculated for all persons as mentioned above, including those with violent crime episodes and those with treatment episodes of injuries caused by violence during the time period. The HR:s for mortality could be somewhat lower than the HR:s in the tables because one or more violent convictions or hospitalizations were not usually reached at 1970, the starting point of the time period.

**Figure 1:**
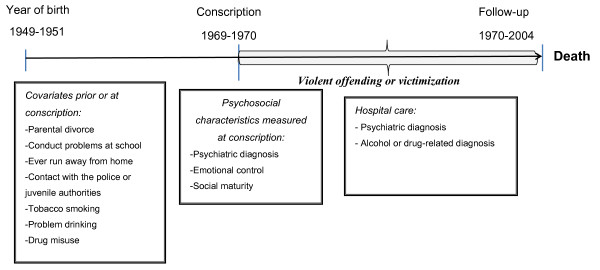
**An illustration of possible predictors of mortality**.

In order to analyze the impact of violence offence, victimization or both on mortality and causes of death, we performed multivariate Cox regression analyses adjusted for early risk factors including alcohol and drug misuse in relation to the outcomes. The early covariates are measured at the time of conscription and the exposure variables (violence, victimization and both) occurred from 1970 and onwards (see Figure 1). In the multivariate models we included only those variables which in the bivariate analyses were significant. We used SAS, version 9.2 (SAS Institute Inc., Cary, NC, USA).

## Results

Of the total cohort, 2,671 (5.5%) died during the follow-up period at an average age of 42 years. Of these, violent offenders died at an average age of 41.8 years, victims 43.2 years and among both victims and offenders 45.3 years. Only a small part (1%, n = 32) died due to violence. Violent offenders (n = 2,139) had a mortality rate of 12.8%, victims (n = 500) of 15.6%, and individuals who were both offenders and victims (n = 235) had one of 22.1%, compared to the mortality rate of 4.9% in the other conscripts (Table 1).

**Table 1:** Underlying causes of death among violent offenders, victims, and individuals who were both victims and violent offenders among 48,834 Swedish conscripts

	Violent offenders N = 2,139		Victims N = 500		Offenders and victims N = 235		Others N = 45,960	
	**Death cases** **n = 274**		**Death cases** **n = 78**		**Death cases** **n = 52**		**Death cases** **n = 2,267**	
	
	**n**	**%**	**n**	**%**	**n**	**%**	**n**	**%**

Accidents*^#^, N = 420	33	12.4	9	11.5	4	7.7	374	16.5
Undetermined suicide*, N = 62	13	4.7	1	1.3	1	1.9	47	2.1
Completed suicide*, N = 425	43	15.7	10	12.8	6	11.5	366	16.1
Alcohol-related^a^, N = 455	95	34.7	29	37.2	20	38.4	311	13.7
Drug-related,**) N = 32	15	5.5	2	2.6	3	5.7	32	1.4
Circulatory disease*, N = 400	34	12.4	11	14.1	7	13.5	348	15.4
Neoplasm*, N = 560	28	10.2	7	9.0	3	5.8	522	23.0
Other causes^ab^, N = 297	13	4.7	9	11.5	8	15.4	267	11.8

^a) ^36 participants comorbid with drug misuse

^b) ^Other causes were: respiratory disease (n = 45), digestive problems (n = 47), endocrine problems (n = 55), a nervous system disorder (n = 57), infection (n = 20), and other diseases (n = 63)

*) Death cases due to accidents, suicide, circulatory, neoplasm, and other causes with also alcohol or drug-related diagnoses were excluded

**) Illicit narcotics-related

*#) Death cases due to accidents were: violence (n = 32), fall (n = 28), traffic (n = 257), other injuries (n = 103)

### Causes of death

The most frequent underlying or contributing cause of death was alcohol-related in all three violent groups (34.7-38.4%, compared to 13.7% in the other conscripts). Even though proportional mortality from suicide was quite similar in all four groups (Table 1), suicide mortality was higher in the violent offender group (2.6%), in victims of violence (2.2%), and in the group of both offenders and victims (3%) compared to the other conscripts (0.9%).

### Bivariate and multivariate analyses

The bivariate analyses showed that the recidivists of violent offences had nearly 11 times higher hazard of dying with an alcohol or drug-related cause and nearly four times higher hazard of suicide compared to the other conscripts, while the victims had six and twofold higher hazards of the same outcomes (Table 2). Death with an alcohol or drug-related cause, and also other causes was high among participants who were both violent offenders and victims: HRs = 10.51 and 6.15, respectively.

**Table 2:** Violence in relation to mortality and underlying causes of death among 48,834 Swedish conscripts

	Accidents N = 420 *HR (95% CI)*	Suicide N = 487 *HR (95% CI)*	Alcohol and drugs N = 507 *HR (95% CI)*	Circulatory disease N = 400 *HR (95% CI)*	Neoplasm N = 560 *HR (95% CI)*	Other causes^a^ N = 297 *HR (95% CI)*	All causes N = 2,671 *HR (95% CI)*
Offenders							
No violent offense	1.0	1.0	1.0	1.0	1.0	1.0	1.0
1 violent offense (n = 1,458)	1.75 (1.13-2.72)	2.03 (1.35-3.07)	4.28 (3.21-5.72)	1.89 (1.21-2.93)	1.05 (0.65-1.70)	0.81 (0.38-1.71)	1.98 (1.68-2.34)
2+ violent offenses (n = 681)	2.21 (1.25-3.93)	3.59 (2.26-5.69)	10.84 (8.23-14.28)	2.68 (1.54-4.66)	1.55 (0.86-2.82)	1.57 (0.70-3.53)	3.94 (3.30-4.70)
Victims (n = 500)							
No victimization	1.0	1.0	1.0	1.0	1.0	1.0	1.0
At least one occasion of being victimized	1.11 (1.02-1.22)	2.29 (1.26-4.16)	6.58 (4.58-9.47)	2.89 (1.59-5.27)	1.38 (0.65-2.91)	3.17 (1.63-6.15)	3.03 (2.42-3.80)
Both offender and victim (n = 235)							
No occasion	1.0	1.0	1.0	1.0	1.0	1.0	1.0
At least one occasion of offending and being victimized	1.20 (1.06-1.37)	3.11 (1.48-6.56)	10.51 (6.92-15.96)	4.03 (1.91-8.50)	1.21 (0.39-3.77)	6.15 (3.05-12.44)	4.37 (3.32-5.74)

Cox proportional bivariate analyses. Hazard ratios (HRs) and 95% confidence intervals (95% CIs) are given in relation to all living participants*

*) Death cases due to accidents, suicide, neoplasm, circulatory disease, and other causes in combination with alcohol or drug-related diagnoses were excluded

^a)^Other causes were respiratory (n = 45) or digestive disease (n = 47), endocrine problems (n = 55), a nervous system disorder (n = 57), infection (n = 20), and other diseases (n = 63)

Multivariate analyses showed high hazards (HR = 2.04 to HR = 3.61) of mortality due to alcohol and drug-related causes in all three groups (Table 3). Moreover, violent recidivists had more than threefold higher hazard of dying from an alcohol or drug related diagnosis and twofold higher hazards of dying from suicide, while no significant risk was found for accidents, neoplasm, other causes and circulatory disease. Having experiences of both violent offense and victimization was especially associated with mortality from other causes (HR = 4.56, 95% CI 2.19-9.47) (Table 3).

**Table 3:** Adjusted*^) ^overall mortality and underlying causes of death**^) ^among violent offenders, victims, and individuals who were both offenders and victims, adjusted for adolescent risk factors in a cohort of 48,834 Swedish conscripts

	Accidents N = 420 *HR (95% CI)*	Suicide N = 487 *HR (95% CI)*	Alcohol and drugs N = 507 *HR (95% CI)*	Circulatory disease N = 400 *HR (95% CI)*	Neoplasm N = 560 *HR (95% CI)*	Other causes^a^ N = 297 *HR (95% CI)*	All deaths N = 2,671 *HR (95% CI)*
Violent offenders							
No violent offense	1.0	1.0	1.0	1.0	1.0	1.0	1.0
1 violent offense	1.26 (0.80-2.00)	1.55 (1.02-2.35)	2.04 (1.49-2.80)	1.48 (0.92-2.38)	0.89 (0.52-1.52)	0.49 (0.20-1.20)	1.39 (1.16-1.66)
2+ violent offenses	1.09 (0.57-2.09)	2.39 (1.51-3.77)	3.03 (2.20-4.17)	1.80 (0.99-3.28)	1.47 (0.79-2.74)	0.85 (0.32-2.12)	2.01 (1.64-2.46)
Victims							
No victimization	1.0	1.0	1.0	1.0	1.0	1.0	1.0
At least one occasion of being victimized	1.10 (1.01-1.21)	2.00 (1.10-3.66)	3.61 (2.41-5.41)	2.69 (1.47-4.92)	1.38 (0.65-2.91)	3.03 (1.56-5.93)	2.45 (1.93-3.10)
Both offender and victim							
No occasion	1.0	1.0	1.0	1.0	1.0	1.0	1.0
At least one occasion of offending and being victimized	1.13 (0.99-3.30)	1.84 (0.86-3.92)	2.57 (1.59-4.17)	2.43 (1.07-5.54)	0.74 (0.18-2.97)	4.56 (2.19-9.47)	2.12 (1.01-1.41)

Cox proportional multivariate analyses. Hazard ratios (HRs) with 95% confidence intervals (95% CIs) are given in relation to all living participants

*)Adjusted for the variables: parental divorce, social maturity, emotional control, contact with the police or juvenile authorities, conduct problems at school, running away from home, smoking, alcohol use, drug use, and psychiatric diagnosis at conscription

**) Death cases due to accidents, suicide, circulatory problems, neoplasm, and other causes in combination with alcohol or drug-related diagnoses were excluded

^a) ^Other causes were respiratory (n = 45) or digestive disease (n = 47), endocrine problems (n = 55), a nervous system disorder (n = 57), infection (n = 20), and other diseases (n = 63)

### Stratification for alcohol, drug and psychiatric inpatient care

In Table 4, we stratified for alcohol, drug misuse, and psychiatric inpatient care during the follow up period. Violent offenders with alcohol or illicit drug diagnoses had nearly fourfold higher hazard to die compared to violent offenders with no such diagnoses. The hazards were much higher when comparing with all other participants: HR = 5.53 and HR = 7.67, respectively. Victims with alcohol-related diagnoses had a nearly fourfold elevated hazard of dying when compared to victims with no alcohol diagnosis, and a more than six fold higher hazard when compared to all the others. Likewise, participants with experiences of both violent victimization and offending and additionally alcohol or drug misuse diagnoses were associated with a nearly six and eightfold higher hazard of dying compared to the others.

**Table 4:** Mortality among violent offenders, victims, and individuals who were both offenders and victims, stratified for hospitalization with an alcohol- or drug-related or a psychiatric diagnosis

	Alcohol inpatient care (only)		
	Violence + alcohol *N*	Violence (only) *N*	Mortality *HR (95% CI)*
Violent offenses + alcohol vs. violence only	596	1,543	3.75 (2.96-4.78)
Victimization + alcohol vs. victimization only	148	352	3.86 (2.46-6.07)
Violence + victimization + alcohol vs. violence + victimization only	146	89	2.45 (1.26-4.77)
	**Drug inpatient care**		
	Violence +drugs	Violence (only)	
	
Violent offenses + drug vs. violence only	206	1,933	3.91 (2.99-5.11)
Victimization + drug vs. victimization only	44	456	1.97 (1.07-3.65)
Violence + victimization + drugs vs. violence + victimization only	65	170	2.56 (1.47-4.42)
	**Psychiatric inpatient care**		
	Violence + psychiatric	Violence (only)	Mortality
	
Violent offenses + psychiatric vs. violence only	463	1676	2.54 (1.99-3.23)
Victimization + psychiatric vs. victimization only	116	384	3.80 (2.44-5.93)
Violence + victimization + psychiatric vs. violence + victimization only	91	144	2.0, (1.58-3.45)
	**Alcohol inpatient care**		
	Violence + alcohol	No violence + alcohol	Mortality
	
Violent offenses + alcohol vs. others	596	48,238	5.53 (4.70-6.50)
Victim + alcohol vs. others	148	48,686	6.45 (4.81-8.61)
Violence + victimization + alcohol vs. others	146	48,688	5.64 (4.14-7.68)
	**Drug inpatient care**		
	Violence + alcohol	No violence + drug	
	
Violent offenses + drug vs. others	206	48,628	7.67 (6.08-9.68)
Victimization + drug vs. others	44	48,790	3.39 (3.06-9.51)
Violence + victimization + drugs			
vs. others	55	48,769	7.74 (5.18-11.57)
	**Psychiatric inpatient care**		
	Violence + psychiatric	No violence + psychiatric	Mortality
	
Violent offenses + psychiatric vs. others	463	48,371	4.81 (3.97-5.83)
Victimization + psychiatric vs. others	116	48,718	7.07 (5.16-9.70)
Violence + victimization + psychiatric vs. others	91	48,743	6.25 (4.31-9.07)

Bivariate analyses using Cox proportional regression. Hazard ratios (HRs) are given with 95% confidence intervals (95% CIs)

Nearly 7% of the total cohort had been treated for a psychiatric disorder. More than one-fifth of the violent offenders and victims also had a psychiatric disorder, which led to higher hazards of dying compared to those without psychiatric disease: HR = 3.80 and HR = 2.54, and the hazards were additionally about twofold higher compared to all others in the cohort.

Considering mortality due to suicide, we also controlled for psychiatric inpatient care alone, and psychiatric inpatient care in combination with alcohol and substance misuse. We found that especially repeat violent offense were associated with higher hazards of suicide, giving an HR of 1.94 (95% CI 1.33-2.82) and 1.71 (95% CI 1.14-2.58), respectively (not shown in tables). No such association was found among victims and individuals who were both offenders and victims.

## Discussion

To the best of our knowledge, this is the first study on mortality and causes of death among violent offenders, victims, or both using a nationwide representative sample with a very long follow-up period as we followed 48,834 men from the age of 18-20 years to about 53 years of age.

One major finding was that the mortality rate was much higher among violent offenders (12.8%), victims (15.6%), and in the offender and victim group (22%) than in the non-violent group (4.9%). Moreover, the alcohol and drug-related causes of death were very high in all three violent groups, while the other conscripts without experience of violence had died more often from neoplasms or accidents. More than one-third of the deaths in each violent group were due to alcohol or drug-related causes, compared to 14% in the non-violent group.

A third finding was that the bivariate analysis showed that having been convicted for repeated violent offense was associated with a nearly eleven fold higher hazard of dying from an alcohol or drug-related cause, while one committed offense resulted in a fourfold higher hazard of dying compared to no offence. Controlling for other risk factors showed that the figures still remained significantly elevated. Further, the fact that about one-third of the violent offenders and victims had been treated for an alcohol-related condition in hospital and about 10% for a drug-related problem means that a large proportion of the offenders and victims suffered from serious substance misuse [4,27-29]. Even though there is a paucity of population-based studies reporting mortality rates due to substance misuse in violent offenders, some studies have shown that alcohol misuse/dependence provides four to eleven times higher risk for violence than abstainers [5,30].

It has been argued that offenders and victims share the same environment including risky behavior like substance use and criminality, as well as mental illness [8,18,31]. Another reason to the similarities in terms of substance use behavior and cause of death between offenders and victims could be that we only captured information on those participants who have been treated in hospital for injuries caused by violence. These participants can be considered severe victims. We consequently lack information about victims who never come to hospital but probably seek treatment on the open ward or elsewhere for violent incidents [22,32].

We found that violent offenders who had also been hospitalized with an alcohol or drug diagnosis had about a fourfold higher risk of dying compared to violent offenders with no substance use. This is in line with results from a large Australian prison cohort study reporting that one third of the death cases were due to alcohol or substance misuse and the standard ratio of drug related mortality after leaving prison was as high as 14.5 [33]. In another Australian study, Darke et al. found, among 400 illicit drug users, that almost all had experienced violent assault during their lifetime and 43% had experiences of self-harm and/or attempted suicide [34]. This implies that alcohol or drug misuse in combination with violent offending elevates the risk of mortality [6,10], why early prevention of alcohol and substance use will probably decrease not only the substance misuse itself, but also the risk of violence and mortality [35].

Some studies have found violence to be more common among persons with psychiatric disorders compared to the general population [36], while results from general population studies revealed that especially psychiatric disorders in combination with alcohol or substance misuse or other risk factors are associated with violence but not psychiatric disorders per se [37-39]. Persons admitted to hospital for a mental disorder had a two to three fold higher mortality than the general population in a recent follow up study from Nordic countries [38]. We found that men with mental disorder and experience of violence either as a perpetrator or as a victim had a two to four fold elevated risk to die compared to those with experience of violence without a mental disorder leading to hospitalization.

Studies have confirmed a strong association between psychiatric disorders and later suicide [12,37,38] and all-cause mortality [5,36]. Suicide has been shown to be a leading cause of death among serious violent offenders [24,40-43]. With regard to repeated violent offenders, analyses showed a nearly fourfold higher hazard to commit suicide, and after controlling for psychiatric inpatient care, the repeaters of violence had about twofold higher suicide risk, indicating that both behaviors share common denominators like behavioral dysregulation of aggression linked to the serotonergic system [43]. A repeated violent offense is an important risk factor for suicide regardless of comorbid psychiatric disorder or substance misuse [42]. Moreover, violent behavior was associated with suicide risk later in life in a recent clinical study of suicide attempters [44].

Our finding suggests that authorities and the health care systems should focus also on prevention of violent behavior, which in turn could prevent future suicide. Further, victimized psychiatric patients had a seven fold higher hazard of dying compared to general population indicating that violent victimization is an important risk factor to target on in the clinical work.

We found, after adjustment for early risk factors, substance misuse and psychiatric disorder at conscription for all the three groups, that the hazards for mortality due to alcohol or drug problems were about threefold higher, while for circulatory and other causes of mortality about a two to fourfold higher risk among victims and individuals with both offences and victimizations but not among the violent offenders (only). In contrast to other studies, the hazards for accidents were very low or non-significant for all the violent groups. It has been reported that especially violent offenders suffer from high impulsivity and risky behavior and are more often involved in various vehicle accidents and other types of accidents leading to serious injuries (1,5).

The participants who had both committed at least one violent crime and been victimized had the highest proportion of death cases (22%) and often also suffered from alcohol (40%) and drug misuse (15%). It has been reported elsewhere that being a victim of violence in itself is a risk factor for offending and vice versa [20]. This violent group probably suffers from other adolescent behavioral and psychiatric problems and more often lives in an environment where criminality and substance use are common [24,45,46]. Among the natural causes of death, neoplasm (23%) was the most common cause among the non-violent participants, which agrees with other general population studies [5].

### Advantages and limitations

One advantage of this nationwide, representative population study is the low non-participation rate, of about 2-3%. The reasons were mainly severe disability or congenital disorders. Use of non-anonymous questionnaires may have resulted in lower reporting of sensitive issues than may have been elicited with anonymous questionnaires. In previous studies of conscripts, although limited to Stockholm, it was found that a large proportion of those participants who had not completed the questionnaire had higher rates of criminality in adulthood. This means that the prevalence of, e.g., drug use may have been underestimated, but it does not mean that the association between the variables is biased [47]. Furthermore, persons with antisocial or paranoid tendencies may be especially prone to deny their use of illicit drugs in non-anonymous questionnaires. Further, we do not know whether the participants had stopped or changed their substance use behavior after conscription, however alcohol or drug-related diagnoses derived from inpatient care registers can be considered a rather more robust measure of alcohol or drug use.

Another limitation of this study is, as has been mentioned above, that we were only able to include victims who had been hospitalized for injuries and we had no information about the perpetrators who had caused the injury. Likewise, information about crimes that had not come to the attention of the police is lacking.

## Conclusions

In conclusion, violent offending and being victimized have an impact on the hazards of especially alcohol and/or drug related mortality and suicide and overall mortality and this relationship is independent from early risk factors including substance misuse and psychiatric diagnosis. It is important to more systematically screen for violent behavior and victimization in clinical settings to detect individuals with high risk. Likewise, in clinical work, patients with experience of victimization or violent offences should be systematically screened for substance abuse and receive comprehensive treatment. Both violent offending and victimization are important to recognize early as they both evince an excess mortality of both natural and unnatural causes.

## Competing interests

The authors declare that they have no competing interests.

## Authors' contributions

TM, AR, JJ and MS designed the study. MS performed the statistical analyses and wrote the first draft of the paper. All authors helped with interpretation of the data and contributed to the drafting, editing and finalizing the paper. All authors read and approved the final manuscript.

## Pre-publication history

The pre-publication history for this paper can be accessed here:

http://www.biomedcentral.com/1471-2458/12/38/prepub
